# Physiological Ripples (± 100 Hz) in Spike-Free Scalp EEGs of Children With and Without Epilepsy

**DOI:** 10.1007/s10548-017-0590-y

**Published:** 2017-09-15

**Authors:** Anne H. Mooij, Renee C. M. A. Raijmann, Floor E. Jansen, Kees P. J. Braun, Maeike Zijlmans

**Affiliations:** 1 Brain Center Rudolf Magnus, Department of Neurology and Neurosurgery, University Medical Center Utrecht, Utrecht University, Heidelberglaan 100, 3584 CX Utrecht, The Netherlands; 2 Faculty of Medicine, University Medical Center Utrecht, Utrecht University, Heidelberglaan 100, 3584 CX Utrecht, The Netherlands; 3 Brain Center Rudolf Magnus, Department of Pediatric Neurology, University Medical Center Utrecht, Utrecht University, Heidelberglaan 100, 3584 CX Utrecht, The Netherlands

**Keywords:** Childhood, High-frequency oscillations, HFOs, Seizures, Surface EEG

## Abstract

Pathological high frequency oscillations (HFOs, >80 Hz) are considered new biomarkers for epilepsy. They have mostly been recorded invasively, but pathological ripples (80–250 Hz) can also be found in scalp EEGs with frequent epileptiform spikes. Physiological HFOs also exist. They have been recorded invasively in hippocampus and neocortex. There are no reports of spontaneously occurring physiological HFOs recorded with scalp EEG. We aimed to study ripples in spike-free scalp EEGs. We included 23 children (6 with, 17 without epilepsy) who had an EEG without interictal epileptiform spikes recorded during sleep. We differentiated true ripples from spurious ripples such as filtering effects of sharp artifacts and high frequency components of muscle artifacts by viewing ripples simultaneously in bipolar and average montage and double-checking the unfiltered signal. We calculated mean frequency, duration and root mean square amplitude of the ripples, and studied their shape and distribution. We found ripples in EEGs of 20 out of 23 children (4 with, 16 without epilepsy). Ripples had a regular shape and occurred mostly on central and midline channels. Mean frequency was 102 Hz, mean duration 70 ms, mean root mean square amplitude 0.95 µV. Ripples occurring in normal EEGs of children without epilepsy were considered physiological; the similarity in appearance suggested that the ripples occurring in normal EEGs of children with epilepsy were also physiological. The finding that it is possible to study physiological neocortical ripples in scalp EEG paves the way for investigating their occurrence during brain development and their relation with cognitive functioning.

**Electronic supplementary material:**

The online version of this article (doi:10.1007/s10548-017-0590-y) contains supplementary material, which is available to authorized users.

## Introduction

High frequency oscillation(s) (HFOs) are oscillations above 80 Hz that are recorded from the brain. They are often defined as four or more oscillations that clearly stand out from the background activity. HFOs are divided in ‘ripples’ (80–250 Hz) and ‘fast ripples’ (250–500 Hz). Both physiological and pathological HFOs exist. Pathological HFOs are considered biomarkers for epilepsy (Zijlmans et al. 2012; Staba et al. 2014; Cimbalnik et al. 2016). Physiological ripples that are recorded in the hippocampus play a role in memory consolidation (Buzsáki 1998; Diekelmann and Born 2010; Maingret et al. 2016). Characteristics of physiological and pathological ripples occurring in people with epilepsy overlap (Matsumoto et al. 2013; Wang et al. 2013; Alkawadri et al. 2014; Von Ellenrieder et al. 2016), which makes distinguishing between the two types of ripples challenging.

HFOs were first recorded with implanted micro-electrodes, but later also with intracranial macro-electrodes (Jirsch et al. 2006; Urrestarazu et al. 2007). The detection of pathological ripples in scalp EEGs with frequent interictal epileptiform discharges or spikes (Kobayashi et al. 2010; Andrade-Valenca et al. 2011) meant that HFO research was no longer restricted to patients undergoing invasive recordings.

Recognizing ripples in scalp EEG is challenging because the amplitude of non-invasively recorded ripples is low and because of the occurrence of muscle artifacts. Muscle artifacts hinder ripple marking in two ways. First, artifacts can obscure the signal, resulting in missed ripples (false negatives). Second, the high frequency component of short muscle artifacts can look like ripples, which, if marked, would be false positives.

The challenges of marking ripples in scalp EEG have been dealt with by selecting recordings with relatively few artifacts and by avoiding most artifacts. Sleep recordings have relatively few artifacts because there is little muscle activity during sleep. Avoiding most artifacts can be done by marking pathological ripples that occur on epileptic spikes, because the search for ripples is then limited to the timeframe of the spike. However, this approach has the disadvantage that all information about ripple activity in traces without spikes is lost.

This study first addressed the question: which settings promote recognizing ripples in spike-free scalp EEG and prevent confusion with spurious ripples such as high frequency components of artifacts? The second question was: do ripples occur in spike free scalp EEG of children with and without epilepsy, and if they do, what are their characteristics? If ripples would occur in normal EEGs of children without epilepsy, we would consider these ripples physiological ripples. If ripples would occur in normal EEGs of children with epilepsy, these ripples could be physiological or pathological ripples.

## Materials and Methods

### Inclusion Criteria

We retrospectively studied EEGs of children (17 years or younger) who had visited our outpatient first seizure clinic because they were suspected of having had one or more (first) seizure(s). We selected all children who fulfilled the following inclusion criteria: (1) EEG recorded at high sampling rate (2048 Hz) and containing at least 10 min of sleep recording, (2) no seizures and no interictal epileptiform discharges or other abnormalities during wake or sleep EEG, according to the clinical reports of specialized epileptologists, (3) no use of anti-epileptic drugs during EEG recording, (4) no structural lesion on MRI (if available), and (5) at least 1 year of follow-up.

Inclusion started in May 2013 because data were routinely sampled at high frequency from that date, and ended in February 2016 to allow a follow-up period of at least 1 year.

We asked informed consent for studying the EEGs and clinical files from parents and from children who were, at the time of the study, 16 years or older. The study was approved by the Medical Research Ethics Committee of the University Medical Center Utrecht who judged that the Dutch Medical Research Involving Human Subjects Act did not apply, provided that data were coded and handled anonymously and informed consent was obtained.

### Follow-Up and Diagnosis

Not all children who visited our outpatient clinic had or developed epilepsy. When we started the study, most children were no longer consulting a pediatric neurologist in our hospital. RR telephoned all parents and children who were over 16 years to collect follow-up data. The minimum follow-up was 1 year. A specialized pediatric neurologist (KB), who was blinded for the results of the ripple marking, determined the diagnosis based on the clinical file and the outcome of the telephone conversation. Children were categorized in two groups: (1) no diagnosis of epilepsy, and (2) diagnosis of epilepsy. Both groups had two subgroups: (1a) no epilepsy and no other brain disorder, (1b) No epilepsy, but another brain disorder (for example migraine or autism), (2a) benign-course epilepsy (for example benign occipital epilepsy syndrome), and (2b) other types of epilepsy (for example frontal lobe epilepsy).

### Data Acquisition

Scalp EEGs were recorded with Micromed Smart Acquisition Module (SAM) and with SD PLUS FLEXI acquisition system (Micromed, Treviso, Italy). Data were sampled at 2048 Hz, low pass anti-alias filter at acquisition was 900 Hz for SAM and 553 Hz for FLEXI. We used conventional 10 mm Ag–AgCl electrodes that were placed according to the international 10–20 system.

### Recognizing Ripples and Avoiding Confusion with Artifacts

We visually marked ripples in Stellate Harmonie (Montreal, Canada). The signal was filtered between 80 Hz (finite impulse response high-pass filter of order 63, as implemented in Harmonie) and 250 Hz (finite impulse response low-pass filter, order 63), amplitude scale was set to 1 μV per mm. We selected sleep recordings and started marking when most artifacts had subsided, even if this was after sleep onset. If short artifacts, for example caused by an arousal or by sudden movements of the head or limps, obscured the signal, we excluded the time-frame of the EEG in which these artifacts occurred for all channels. In this way, all obvious artifacts were discarded.

We discovered that it was easiest to recognize ripples and to prevent confusion with spurious ripples such as high frequency components of artifacts when we split the window vertically and viewed the high frequency signal simultaneously in bipolar (double banana) and average montage. Time scale of both windows was 0.4 s per page. The advantage of viewing the signal in average montage was that ripples often stood out more clearly from the background activity compared to the same event viewed in bipolar montage. However, a disadvantage of the average montage was that an artifact recorded by one electrode, for example T7, could in an average montage give rise to spurious ripples on several channels, even those located far from the electrode with the artifact. This problem did not occur in a bipolar montage: an artifact in T7 was visible in channels F7-T7 and T7-P7 only. The bipolar montage was therefore important to determine if the ripple that was spotted in the average montage was false or real. We also checked the signal below 80 Hz in both montage to make sure the event was no muscle artifact or filtering effect. If we would remain doubtful if a ripple was real despite these precautions, we did not mark the ripple.

We marked ripples in both montages and in all channels. Events were marked as ripples if they consisted of four or more oscillations that clearly stood out from the background activity. If background low-amplitude ripple band activity built up to visually recognizable ripple oscillations, we started marking when the oscillations stood out clearly from the overall background activity of the channel. When the time-window of a ripple partly overlapped with that of a ripple on another channel, we marked both ripples. Ripple marking was done by AM and checked by MZ.

### Cumulative Ripple Rate

We used ripple rate per minute, calculated over a period of 10 min, as a measure of frequency of ripple occurrence. We first summed the number of ripples occurring in all channels per minute. If the sleep recording was longer than 10 min, we selected the ten consecutive minutes in which the maximum number of ripples occurred. The actual time available for marking ripple during those 10 min might be shorter because of the occurrence of artifacts. Thus, cumulative ripple rate per minute was calculated by taking the total number of ripples occurring in the 10 min with the maximum number of ripples for that child and dividing it by 10 min minus the duration of artifacts during those 10 min.

### Ripple Characteristics and Distribution

Ripples are marked in bipolar montage in almost all HFO studies. We calculated frequency, duration, and root mean square amplitude of ripples marked in bipolar montage to enable comparison with these characteristics reported in other studies. Frequency was calculated from the number of zero crossings, root mean square amplitude was computed as the square root of the average power. All ripple characteristics were calculated in filtered (80–250 Hz) data using Matlab (version R2015b, The MathWorks Inc., USA.).

We counted the number of ripples per channel and summed the number of ripples per channel of all children for a general overview of the distribution of these events.

## Results

### Characteristics of Included Children and Occurrence of Ripples Across Diagnostic Groups

Twenty-six children met the inclusion criteria. We obtained informed consent for using the EEGs and clinical data for 23 children (14 boys), aged 11 months to 14 years at the time of the recording. Ten children had no epilepsy, and no other brain disorder (category 1a). Seven had no epilepsy, but another brain disorder (category 1b): there were two children with migraine, two children with autism spectrum disorder, one child with a chromosomal microduplication and autism, one child with GLUT1 (Glucose Transport type 1) deficiency syndrome, and one child with a history of acute symptomatic seizures during bacterial meningitis. Three children had benign-course epilepsy (category 2a), and three children had other types of epilepsy (category 2b). We found ripples in the EEGs of 20 out of 23 children: in 9 out of 10 EEGs of children in category 1a, in all EEGs of children in category 1b and 2a, and in 1 out of 3 EEGs of children in category 2b. Characteristics of included children are provided in Supplementary Table 1.

### Ripple Rate

The longest sleep recording was 47 min, the shortest 10 min. Most events occurred in the first 10 min of the sleep recording in most children (16 out of 20 children). In two children, most ripples occurred in 10 min that partly overlapped with the first 10 min; in the other two children, most ripples occurred in completely different 10 min epochs.

**Fig. 1:**
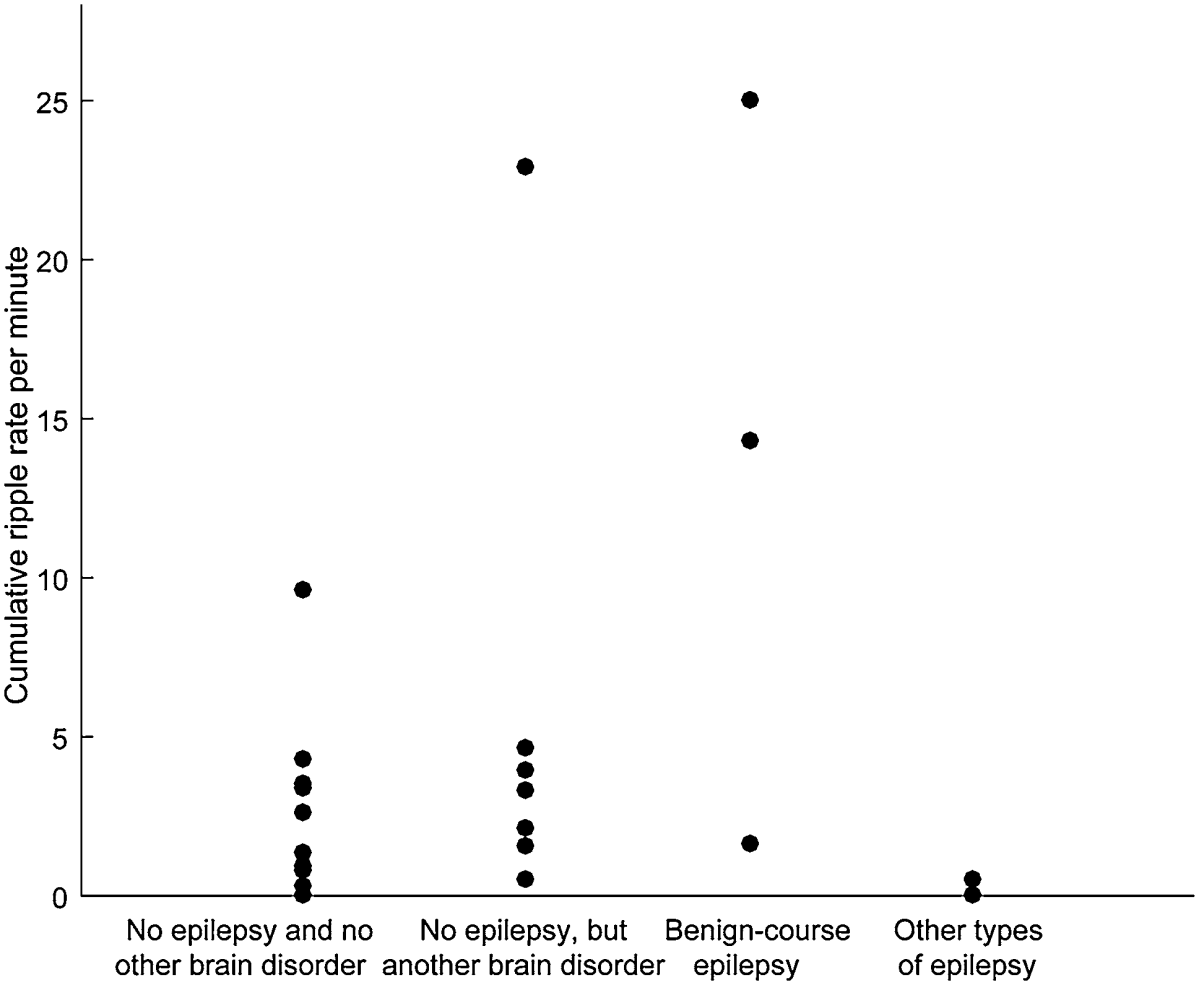
Plot of the ripple rate per child in each diagnostic category. Cumulative ripple rate per minute was calculated by taking the total number of ripples (in the 10 min with the maximum number of ripples for that child) and dividing it by 10 min (minus the duration of artifacts during those 10 min). Ripples occurred across all diagnostic categories

Cumulative bipolar ripple rate ranged from 0.0 to 25.0 ripples per minute, ripple rate in most children was below 5 ripples per minute (Fig. 1; Supplementary Table 1).

### Ripple Characteristics

**Fig. 2:**
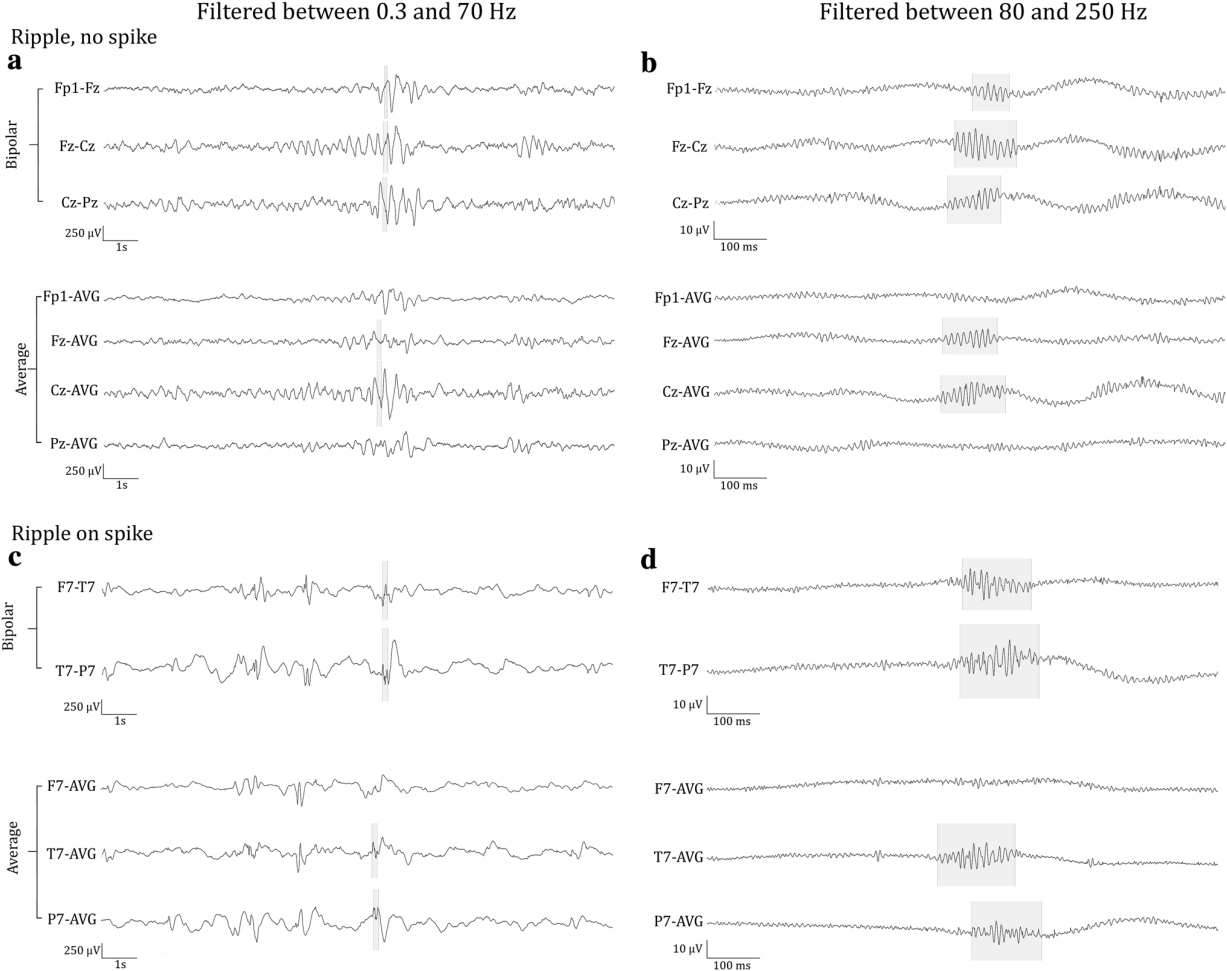
Examples of ripples marked in this study (**b**), which do not occur on spikes (see corresponding grey area in (**a**) and of ripples (**d**) that occur on epileptic spikes (see corresponding grey area in (**c**). The EEG with epileptic spikes was not included in this study, the examples are shown for comparison with the ripples shown in (**b**). The EEG traces in **a** and **c** are filtered between 0.3 and 70 Hz, time scale is 15 s per page, amplitude scale is 30 µV per mm. The EEG traces in **b** and **d** are filtered between 80 and 250 Hz, time scale is 1 s per page, amplitude scale is 1 µV per mm. Channels are shown in bipolar and average montage. Note the difference in appearance of the regular ripples in **b** and the more irregular ripples in **d**

Ripples had a regular shape (Fig. 2b), more so than ripples on epileptic spikes (Fig. 2d). The EEG with spikes, illustrated in this figure, was not included in this study; the examples of ripples on spikes are provided for comparison with the examples of ripples marked in this study. Examples of the corresponding low frequency signal is provided in Fig. 2a, c, examples of EEG traces containing both low and high frequencies is provided in Supplementary Fig. 1.

Median frequency ranged from 91 to 116 Hz. The mean of the 20 medians was 102 Hz. Median duration ranged from 55 to 85 ms, the mean of medians was 69 ms. Root mean square amplitude ranged from 0.76 to 1.39 µV, mean of medians was 0.95 µV. An impression of the amplitude of the ripples can be obtained from Fig. 2b.

### Ripple Distribution

**Fig. 3:**
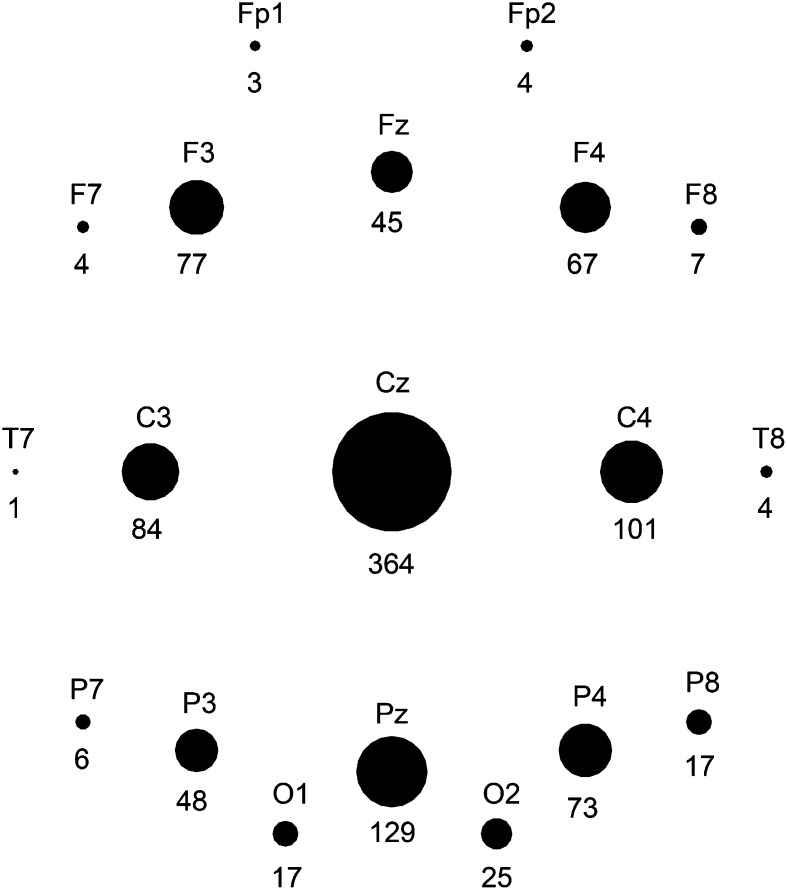
Schematic overview of the number of ripples per channel, marked in average montage. Number of ripples is written underneath the black circle that represents the channel, and reflected in the size of the black circle. Ripples occurred most frequently in central and midline channels, particularly Cz

Total number of ripples per child ranged from 4 to 364 for channels in bipolar montage, and from 4 to 238 for channels in average montage. We summed the number of events per channel of all children and found that most events occurred on Cz-Pz (374 ripples, 26% of total number of ripples marked in bipolar montage), Fz-Cz (224 ripples, 16%), and Pz-O2 (139 ripples, 10%). For the average montage, the three channels with most ripples were Cz (364 ripples, 34% of total number of ripples marked in average montage), Pz (129 ripples, 12%), and C4 (101 ripples, 9%). The average montage seemed the more precise montage for studying the localization of ripples. For example, Fig. 2b shows that ripples occurring on bipolar channels Fp1-Fz, Fz-Cz, and Cz-Pz are only visible on Fz and Cz in the average montage. Distribution of all ripples marked in average montage is shown in Fig. 3.

### Ripple Rate and Age

**Fig. 4:**
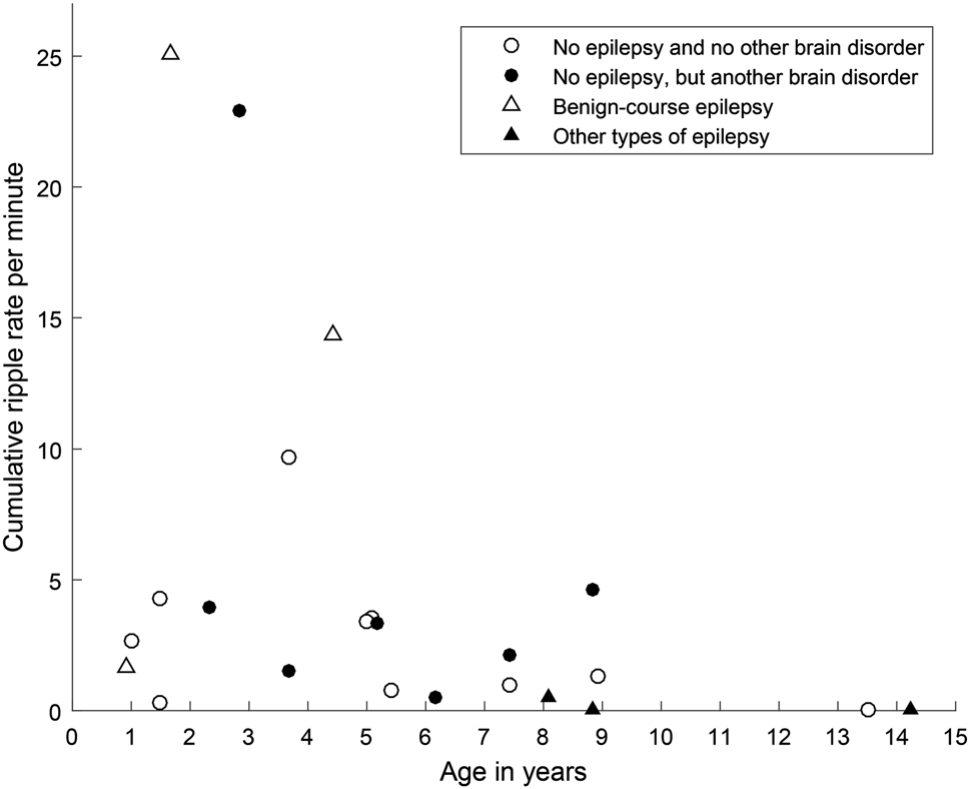
Scatterplot of age versus cumulative ripple rate. Ripple rates of children without a neurological diagnosis are plotted as open circles, ripple rates of children without epilepsy, but with another brain disorder as closed circles. Ripple rate of children with benign-course epilepsy are plotted as open triangles, ripple rate of children with other types of epilepsy as closed triangles

The choice to study children was a practical one: recording at high sampling rate started in our hospital in this cohort of children. We had no specific hypothesis about a relation between ripple rate and age, but noticed that high ripple rates seemed to occur particularly in young children. We plotted the data to get an impression if this might be a topic that would merit future research in a bigger cohort. A scatter plot with age on the x-axis and cumulative ripple rate on the y-axis showed that high ripple rates occurred in children under five, and the oldest two children, who were 13 and 14 years old when the EEG was recorded, had no ripples (Fig. 4). This suggested there might be an age-related pattern. On the other hand, most children between 1 and 9 years old had ripples rates between 0 and 5 ripples per minute, without any clear age-related trend.

## Discussion

This HFO study showed that it is feasible to mark ripples in spike-free scalp EEGs. Comparing the occurrence, distribution and shape of ripples in bipolar and average montage, as well as double-checking the unfiltered signal in both montages, promotes recognizing ripples and prevents confusion with spurious ripples such as high frequency components of artifacts.

Ripples occurred in 16 out of 17 normal EEGs of children without epilepsy. These ripples were considered physiological ripples. They had a regular shape; their appearance differed from the shape of pathological ripples that occur on spikes.

Ripples also occurred in four out of six normal scalp EEG of children with epilepsy. These ripples had the same regular shape as ripples marked in the EEGs of healthy children. We therefore think that the ripples marked in EEGs of children with epilepsy were also physiological. Another reason to think these ripples were physiological is that pathological ripples are probably rare in spike-free scalp EEG. A study that looked at the occurrence of pathological ripples in EEGs with different spike rate found that there were fewer pathological ripples when spike rate was lower (Melani et al. 2013). Melani et al. (2013) also included five EEGs without spikes; they found a few pathological ripples in only one of them. Two of the three children with benign-course epilepsy (category 2a) included in this study had high ripple rate (Fig. 1); it seems unlikely that so many pathological ripples should occur in spike-free EEGs. As for category 2b: two of the three children in this category had no ripples, the third, with epilepsy of unclear classification, had a ripple rate of 0.5 (i.e., 5 ripples in 10 min). Because of their regular shape and their occurrence in normal background, we think it unlikely that these ripples were pathological, but we cannot rule out this option completely.

Frequency and duration of ripples presented in this study were similar to those reported in previous studies on characteristics of physiological ripples (Wang et al. 2013; Alkawadri et al. 2014; Von Ellenrieder et al. 2016). Amplitude was not comparable, which is inherent to the fact that those studies used invasive recording techniques. Ripple rate was comparable to the rate reported by von Ellenrieder et al. (2016).

Comparing distribution is complicated because electrode localization differs per patient when using invasive recording techniques. However, the centrally located ripples reported in this study might be the same type of oscillations that Wang et al. (2013) found in the primary motor cortex. Our results differ from those of Alkawadri et al. (2014), who reported that highest rates of non-epileptic ripples occurred in occipital contacts. We can think of two reasons why the number of occipital ripples might have been underestimated in the present study. First, most ripples occurred on central and midline channels. It is therefore plausible that the number of ripples would have been higher in an Oz channel (or Pz-Oz, in a bipolar montage) than in (Pz-)O2 or (Pz-)O1. The second reason is that if people sleep lying on their back, most artifacts in scalp EEG occur in occipital channels. The chance of finding spurious ripples was therefore higher in these channels. This had no effect on the procedure for marking ripples, which was the same as in other channels, but we were especially cautious when marking ripples in occipital channels, and as a result we might inadvertently have discarded some true ripples.

We could not find any studies on spontaneously occurring physiological high frequency oscillations recorded with scalp EEG. In the field of cognitive neuroscience most reports focus on high frequency spectral power that is evoked or induced by a stimulus or task. This approach has the advantage that one knows when to expect the high frequency activity, and challenges of artifacts can sometimes be dealt with by averaging the evoked response. Spontaneously occurring high frequency activity that was visible on time–frequency plots has also been reported. Menicucci et al. (2013) recorded activity up to 125 Hz in healthy sleeping adults and Chu et al. (2014) found activity up to 95 Hz in healthy sleeping children of 0–18 years. However, neither of these studies provided detailed information about oscillations, and, as Buszáki and Lopes da Silva (2012) wrote: “…increased power in a given band does not warranty the presence of an oscillation”.

The figure of ripple rate and age showed no clear age-related pattern, but there might be a trend of a decreasing ripple rate in older children. The previously mentioned study of children of 0–18 years reported that high frequency power increased steadily after 5 years, most prominently in the central regions (Chu et al. 2014). Although this central distribution matches the distribution found in our study, Chu et al. (2014) might have reported about different high frequency activity. They studied a wider and lower frequency band (50–100 Hz) than our observed frequencies (91–116 Hz) and we cannot retrieve any details of the oscillations, because the authors provided only time–frequency plots. However, these potentially conflicting findings suggest that the development of physiological high frequency oscillations in children is a relevant topic for future research. Future studies are also needed to test if these ripples occur in adults.

Can these physiological ripples be related to the hippocampal physiological ripples that play a role in memory consolidation? We know of no studies that show a direct link between physiological ripples in the hippocampus and neocortex, but the HFO literature suggests that hippocampal and neocortical ripples might be connected through sleep oscillations such as spindles and slow waves. Several papers report that the physiological ripples occurring in the hippocampus are linked to such sleep phenomena (for example, Diekelmann and Born 2010; Maingret et al. 2016), and coupling between neocortical ripples and sleep phenomena was shown in healthy animals (Grenier et al. 2001; Averkin et al. 2016) and in people with chronic epilepsy and implanted electrodes (Frauscher et al. 2015; Nonoda et al. 2016; Von Ellenrieder et al. 2016). While checking the unfiltered signal, we noticed that the ripples reported here also seem to co-occur with sleep phenomena. Our first impression was that ripples were not limited to spindles and slow waves, but also occurred during sharper physiological transients such as vertex waves and hypnagogic hypersynchrony. Our next step will be to do a quantitative analysis of the co-occurrence of ripples and sleep oscillations. The fact that we study spike-free EEGs might be advantageous for investigating the relation between physiological ripples and sleep phenomena, as interictal spikes were shown to affect sleep patterns (Gelinas et al. 2016).

## Limitations

A limitation of this study is the small number of subjects. Unfortunately, such a small sample size is hard to avoid. The required high sampling rate limited the period in which children could be included, because routine recording at high sampling rate started in 2013. Moreover, normal sleep EEGs were rare, particularly in children with epilepsy. Of the 315 children with an EEG recorded at high sampling rate, only 26 fulfilled all the inclusion criteria.

A second limitation is that the normal EEGs were obtained in a clinical setting. None of the children in this study suffered from chronic epilepsy when the EEG that was analyzed in this study was made, and ten children had no neurological diagnosis after a follow-up period of at least 1 year. The similarity in appearance of ripples occurring in normal EEGs of children across diagnostic groups, including healthy children, suggests that the ripples described in this study are physiological. We can, however, not rule out the option that the ripples found in the children with a brain disorder other than epilepsy reflect some kind of pathological, albeit not epileptic, events. We hope that the finding that it is possible to study ripples in normal scalp EEG will inspire research on spontaneously occurring HFOs in healthy participants who have never been suspected of having any neurological or psychiatric disorder. Such studies are also needed to investigate the relation of these ripples to physiological brain functions.

## Conclusion and Future Directions

This is the first study to report spontaneously occurring physiological ripples recorded with scalp EEG. The next step is to investigate their occurrence across sleep stages and their co-occurrence with sleep oscillations. Future studies should clarify if these ripples occur only in children, with a potential relation to brain development, or can also be found in adults.

## Electronic supplementary material

Below is the link to the electronic supplementary material.

Supplementary Figure 1: Examples of EEG traces containing low and high frequencies. This figure shows the same traces as in Fig. 2a (bipolar channels), but the low pas filter is 250 Hz instead of 70 Hz. Time scale is 15 seconds per page, amplitude scale is 30 µV per mm. An enlargement of the grey area containing the ripple marking (see also Fig. 2b) is shown below each trace. (TIF 4003 KB)

Supplementary Table 1: Characteristics of included children (DOCX 25 KB)
