# Supplementary figures and images for: Physiological Ripples (± 100 Hz) in Spike-Free Scalp EEGs of Children With and Without Epilepsy
Source: Brain Topogr. 2017 Sep 15;30(6):739–46. doi: 10.1007/s10548-017-0590-y (PMC5641281; doi:10.1007/s10548-017-0590-y)

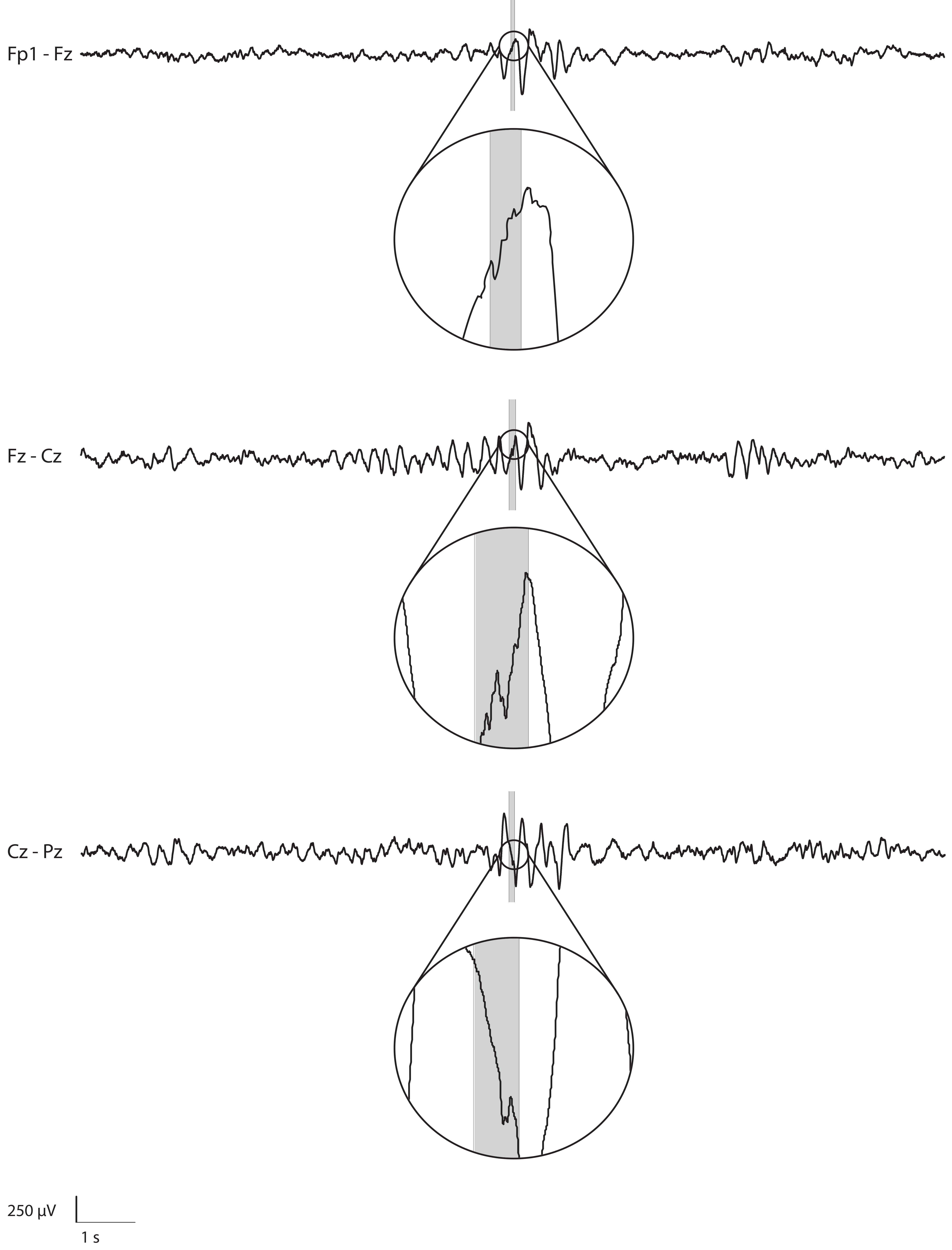

Supplement: Supplementary file 1 — Supplementary Figure 1: Examples of EEG traces containing low and high frequencies. This figure shows the same traces as in Fig. 2a (bipolar channels), but the low pas filter is 250 Hz instead of 70 Hz. Time scale is 15 seconds per page, amplitude scale is 30 µV per mm. An enlargement of the grey area containing the ripple marking (see also Fig. 2b) is shown below each trace. (TIF 4003 KB) [file 10548_2017_590_MOESM1_ESM.tif]
