# Supplementary material for: Physiological Ripples (± 100 Hz) in Spike-Free Scalp EEGs of Children With and Without Epilepsy
Source: Brain Topogr. 2017 Sep 15;30(6):739–46. doi: 10.1007/s10548-017-0590-y (PMC5641281; doi:10.1007/s10548-017-0590-y)
Supplement: Supplementary file 2 — Supplementary Table 1: Characteristics of included children (DOCX 25 KB) [file 10548_2017_590_MOESM2_ESM.docx]

| Sub-ject num-ber | Gen-der | Age at time of sleep EEG | Ripple rate per minute^1^ | Duration follow-up | Diagnosis | Diagnostic  category^2^ |
| --- | --- | --- | --- | --- | --- | --- |
| 1 | M | 1y8m | 25.0 | 3y7m | Probably benign childhood epilepsy variant, exact classification unclear | 2a |
| 2 | M | 3y8m | 1.6 | 3y4m | Glut 1 deficiency syndrome, no epilepsy | 1b |
| 3 | M | 14y3m | 0.0 | 3y4m | Idiopathic generalized epilepsy with nocturnal GTCS | 2b |
| 4 | F | 13y6m | 0.0 | 3y3m | No epilepsy, no diagnosis | 1a |
| 5 | M | 2y4m | 3.9 | 3y5m | Autism | 1b |
| 6 | M | 1y6m | 4.3 | 3y3m | No epilepsy, no diagnosis | 1a |
| 7 | M | 5y1m | 3.5 | 3y3m | No epilepsy, possibly sleep disorder | 1a |
| 8 | M | 2y10m | 22.9 | 3y1m | No epilepsy at time of follow-up, in the past acute symptomatic seizures during bacterial meningitis | 1b |
| 9 | M | 3y8m | 9.7 | 3y0m | Diagnosis unclear, epilepsy highly unlikely | 1a |
| 10 | F | 5y0m | 3.4 | 2y9m | No epilepsy, no other diagnosis | 1a |
| 11 | F | 7y5m | 1.0 | 2y9m | No epilepsy, tics | 1a |
| 12 | M | 8y11m | 1.3 | 1y9m | No epilepsy, no other diagnosis | 1a |
| 13 | M | 8y10m | 4.7 | 2y7m | No epilepsy, tic syndrome, and  autism spectrum disorder | 1b |
| 14 | F | 7y5m | 2.1 | 2y6m | CACNA1A deficiency, migraine, no proof of diagnosis epilepsy | 1b |
| 15 | F | 8y10m | 0.0 | 2y0m | Frontal lobe epilepsy of unknown cause | 2b |
| 16 | M | 5y2m | 3.3 | 2y0m | No proof of epilepsy, chromosomal microduplication syndrome and autism | 1b |
| 17 | F | 0y11m | 1.7 | 2y1m | Benign infantile epilepsy syndrome | 2a |
| 18 | M | 1y6m | 0.3 | 2y0m | No epilepsy, no diagnosis | 1a |
| 19 | F | 4y5m | 14.3 | 1y9m | Benign occipital epilepsy syndrome | 2a |
| 20 | M | 1y0m | 2.7 | 1y7m | No epilepsy, no diagnosis | 1a |
| 21 | F | 6y2m | 0.5 | 1y3m | Migraine, epilepsy not definitely excluded | 1b |
| 22 | F | 8y1m | 0.5 | 1y0m | Epilepsy, unclear classification | 2b |
| 23 | M | 5y5m | 0.8 | 1y0m | No clear epilepsy, no other diagnosis | 1a |

^1^ Calculated by taking the total number of ripples (of all channels in the 10 minutes with the maximum number of ripples for that child) and dividing it by 10 minutes (minus the duration of artefacts during those 10 minutes).

^2^1a) No epilepsy and no other brain disorder; 1b) No epilepsy, but another brain disorder; 2a) Benign-course epilepsy; 2b) Other types of epilepsy

GTCS: Generalized tonic-clonic seizures
